# Pyorrhea Alveolaris

**Published:** 1905-10-15

**Authors:** C. A. Burbridge

**Affiliations:** Grand Rapids, Mich.


### PYORRHEA ALVEOLARIS.

---

BY C. A. BURBRIDGE, D.D.S., GRAND RAPIDS, MICH.

---

Read before the Michigan State Dental Association, July 11, 1905.

---

It has only been during recent years that the disease known as pyorrhea alveolaris has been differentiated as a separate and distinct malady from other inflammatory conditions affecting the oral cavity. It's etiology is even now conceded by the broader minded dental pathologists to be more or less vague and undetermined. The theories that have been advanced both in this country and abroad, are almost numberless. However, the careful study and research on the subject have elicited facts which avail us in coming to a more definite decision concerning it.

The etymology of the term pyorrhea alveolaris is significant and yet does not give us any hint as to the cause or nature of the disease. "Pyon," from the Greek, meaning pus, and "rheo," also Greek, meaning "I flow" make up the first part of the term pyorrhea. "Alveolaris" of course refers to the alveolus. Thus we have—pus flowing from around the alveolus. This, however, is merely one of the symptoms of the disease to which our attention may be called either by the patients telling us, or subsequent to examination of the oral cavity.

The term pyorrhea alveolaris is conceded to be descriptive of degenerative conditions which are characterized by the progressive loosening of teeth in their sockets, accompanied by a loss of the retentive alveolus and pericementum, pus oozing in the majority of cases from around the loosened teeth, and deposits of calculi around the exposed roots. Hence we see the inadequacy of the name as a key to its distinctive characteristics.

Many pathologists and others interested in the development of satisfactory knowledge concerning the disease have attempted the classification of all the forms of tooth-loss, characterized by the above pathological features, under one head, based upon pathogenesis. This, however, has failed, as has also the separation of a specific bacterium as a causative element in the production of pyorrhea. It is not improbable that the untiring efforts of scientific men will eventually effect this, as Kirk, and Miller have already found by repeated analytical tests, the presence of the diplococcus pneumoniae in a large percentage of exudates from the inflammatory conditions accompanying pyorrhea. Hence we obviously include local infection in the list of local causes of this condition.

The other elements entering into a classification of local causes are, the local and general causes of marginal and interstitial gingivitis, and calcarious deposits. Films collecting around and about the neck of the teeth form excellent lodgement for the growth and proliferation of bacteria and these then infect the degenerated tissues of gum and pericementum, which have become so degenerated by the hyperæmia or inflammation caused, as Burchard says, "by the overuse, abuse or disuse of the teeth." This condition is also included under local causes of marginal gingivitis, the truth of this being proved to us, when upon removal of these bacterial plaques, the inflammatory condition ceases. Another local cause of marginal gingivitis which may subsequently cause pyorrhea is mechanical irritation. Under this we have a large classification.

It may include deposits of calculi about the tooth necks, rough margins of cavities not filled at the cervical aspect of a tooth, overhanging margins of fillings, ill-fitting crowns for bridge abutment, collections of food-materials which undergo subsequent fermentation, use of metal tooth picks, harsh tooth brushes and ligatures, pieces of wood tooth picks left between the teeth, also catarrhal conditions due to excessive smoking and free imbibing of alcoholic liquors.

The general causes of marginal gingivitis which, as has been stated, tend toward the production of pyorrheal conditions are malnutrition and auto-intoxication. Substances of an irritating nature such as normal or abnormal waste, metabolic products are retained in the blood current. Nature endeavors to eliminate these by way of the gums and pericementum, and this naturally causes an irritation resulting in debility and the susceptibility toward bacterial infection. Mouth infection results in intestinal infection and hence we have fermentative processes going on—the formation of toxins and their subsequent absorption, resulting in the condition—auto-intoxication.

Interstitial gingivitis also furnishes one of the causative elements of pyorrhea. The condition itself is an inflammation due to an excessive number of leucocytes in the connective tissue elements of the pericementum, attracted there by local irritants or by the waste products of auto-intoxication. It is characterized by a long continued low grade of inflammation resulting in resorption or hypertrophy of bone, resorption or hypertrophy of cementum, or both combined.

This resorptive process which takes place is of three varieties, the first being that of the lacunae which is caused by a proliferation of surface osteoblasts that excavate depressions known as "Howship's Lucunae." These deepen and widen to the extent of complete alveolar extermination. The second class begins in Volkmanns' canals, which are minute connections between the medullary spaces. Here the resorptive process begins and works its destructive katabolism. The third process is known as "halisterisis ossium," a condition in which the calcium salts, especially the phosphates, are lost leaving only the gelatinous matrix, which is itself subsequently absorbed finally resulting in the condition "osteomalacia." Talbot states in connection with this, that chronic irritation such as waste irritative products would cause, tend to lessen the lumen of the blood vessels, impeding the blood flow and resulting in impaired nutrition of a part. This condition is termed "endarteritis obliterans."

Another factor which presents itself as one of the causes of pyorrhea is the deposition of calculus beneath the gum margin, commonly known as sub-gingival calculus. This I might say, is probably the most important exciting cause. We understand by the word "calculus" that it is a deposition of calcium salts mixed with an altered marginal secretion—probably mucin. Just how this combination takes place is difficult of comprehension, but it is noted that if from any cause an excess of salivary secretion is induced, the gaseous carbon dioxid which holds the calcium in solution volotilizes and causes a precipitation of the calcium carbonate and phosphate from the saliva and this becomes entangled in the mucoid constituents, together with fermented food substance and bacterial organisms. This mass takes on the hardened character after having been forced into the spaces between the gums and tooth necks. There are three separate and distinct varieties of calculus, but only one, the sub-gingival variety, which is of importance in connection with the subject in hand. This particular variety of calculus is characterized by its usual dark color, its hardness, and also its particular affinity for tooth structure—this fact often rendering its removal exceedingly difficult. These deposits vary as to surface texture, they being sometimes rough and sometimes smooth. As a rule their form is disc shaped—the deposit assuming a circular form.

The presence of these deposits beneath the gum margins in contact with the mucous membrane necessarily constitutes a local mechanical irritant. The extent to which this irritation may go of course depends upon the comparative roughness or smoothness of deposit, and to the predisposition to pericemental degeneration. However, the inflammation, greater or less as it may be, finally results in gum resorption together with alveolar resorption and the successive deposits of calculus may result in complete extermination of tooth retention by the various forms of resorption before described. These deposits never occur on the enamel faces, but instead upon cementum. Perforce it is only when these deposits have brought about degenerative changes in pericementum sufficient to allow bacterial infection and subsequent pus formation that we have the real pyorrhea condition existant.

General systemic conditions enter into a consideration of the causative elements of pyorrhea alveolaris from the fact of their bringing about a general degenerated condition of the pericemental tissues, thus rendering them more susceptible to the irritative action of calculi and other mechanical irritants. Arthritic diseases are especially prone to production of such conditions, as are also anæmia, leukæmia and diabetes, all of these having degenerative influences upon the pericementum.

Clinically speaking the disease pyorrhea may be divided into two grand classes of varieties, namely, first—those cases associated with marginal gingivitis and accompanied by subgingival calculus, resorption of gum tissues, pericementum and alveolus, and pus formation. Second, those cases in which marginal gingivitis is not perceptible, but which are characterized by pericemental degeneration and necrosis, formation of secondary abscess, and complete dissolution of tooth retention. Dr. Black is responsible in a large measure for the conclusive research on this particular phase of the disease and has very appropriately named it "phagadenic pericementitis." This variety is confounded diagnostically by many with gouty pericementitis, although its causes are altogether separate and distinct.

The causes of the first class may be divided into predisposing and exciting; the predisposing causes including any conditions which give rise to subacute inflammation of the gingivae such as marginal gingivitis, and also such debilitating influences as overuse, misuse or disuse of the teeth; the exciting causes including mainly presence of subgingival calculus and infection of irritated tissues.

If this stage of the disease is seen, early collections of fermenting food substances and filmy attachments will be found at the tooth necks and a pocket existing between gum and tooth. This does not become marked, however, until the deposits have encroached upon the alveolar margins considerably, the gum margins becoming more swollen, the periosteum undergoing necrotic changes and the alveolar wall in a state of dissolution. Pus forms in the pocket beneath the calcareous deposit thus involving all the tissues beneath in a suppurative degeneration. The deposits advance upon alveolus and pericementum—the progress of the disease increasing with rapidity until the tooth is gradually denuded. Rarely during the process is the tooth particularly sore unless for some reason the pus is confined. It is noticeable that the teeth are strangely free from carious processes during the progress of these other degenerative changes. A probe will generally disclose the nature of the bone resorption whether due to atrophic changes or osteomalacia. B'ack has called our attention to an interesting condition which is sometimes found, namely, a constructive process taking place on the outside of the alveolar plate at the same time as the atropic process is taking place in the socket; this being due to the diminishing degree of inflammation from within outward reaching a stage of mild irritation and bringing about constructive changes.

This variety of pyorrhea is attended with but little difficulty in diagnosis. As a rule several teeth are affected simultaneously. The marginal inflammation, pus oozing upon pressure being applied to the gums, and the presence of subgingival calculus all tend to distinguish the condition from any other oral infection. Not infrequently the odor from such a condition is diagnostic also.

In the consideration of the treatment of this condition let me state as a prefice that prophylaxis is the prime and all-important factor. If, after the condition is well in hand, a frequent and regular removal of all calcareous deposits and other disease causes is not practiced, relapse is sure and inevitable.

I shall preceed the medicinal treatment of this variety of pyorrhea by a brief review of the methods for correction of extreme mobility, since it is obvious that in case of degenerations of pericementum and alveolus have reached a state when tooth retention is practically lost and extreme mobility is present, some means for prevention of this must be resorted to before even thorough scaling may progress. To accomplish this we resort to the various forms of splints —the character and advisability of which depend entirely upon the nature of the case. Many times the mere ligating of the teeth temporarily with floss silk or brass wire is sufficient to aid in the scaling process and also assist in the tooth regaining firmness in socket. Brass wire is especially well adapted from the fact of its strength and pliableness, and it is claimed by some to have antiseptic properties similar to copper amalgam. In order to keep silk ligatures in place small buttons of zinc phosphate may be placed upon the labial faces of the teeth after having thoroughly dried them. The ligatures may be saturated with a solution of celluloid in acetone to prevent disintegration. Another form of temporary splint which is often available is the use of a thin platinum band, swaged to accurately fit the lingual surfaces of the teeth, and this held in position by wire ligatures. A platino-irridium bar may be likewise used, and retained in the same manner.

Temporary splints are, however, not always sufficient to meet the requirements of the case and we are then compelled to resort to permanent splints. These are many, and varied in type, some of the appliances used in tooth regulating offering admirable means of retention. If but two or three teeth anteriorly are to be splinted, rings of thin platinum plate soldered together and cemented to the teeth answer the purpose and also have the advantage of eliminating pulp devitalization. The Smith devise offers an admirable method providing the patient will submit to pulp devitalization (which I might here state is often of advantage in early stages of pyorrhea by directing the entire blood supply to the pericementum thus providing added nourishment to that part.)

The devise consists in enlargement of canal orifices (after having divitalized the pulp), on the lingual surfaces of the affected teeth and swaging a piece of thin gold or platinum plate to accurately fit these surfaces. Pins fitting the root canals are pushed through the plate into the canals, an impression taken, and the whole invested and soldered. Having dried the root canals and surfaces of the teeth, the appliance is cemented into place. This appliance shows no metal labially and hence is advantageous.

Another form of splint was introduced by Dr. M. L. Rhein which is equally well adapted for either anterior or posterior teeth. It, however, necessitates devitalizing the pulp as do most of the permanent appliances. It consists in making a groove in the teeth—if anterior on the lingual surfaces, if posterior on the occlusal surfaces—and fitting a piece of triangular iridio-platinum wire in the groove. To this are soldered pins which pass into each root canal. The appliance is cemented into position, but before the cement hardens, small pieces of gold foil are carefully packed into it. It is then allowed to harden after which more gold is packed in around the bar, the groove entirely filled and the whole finished down as is a gold filling. Amalgam could be substituted for gold in many cases, answering the requirements.

In the May *Cosmos* for 1903, an article appeared by Dr. Ames in which be described a rather ingenious permanent splint, applicable mainly to anterior teeth. In brief it was as follows: Pulp devitalization was followed by crown amputation of the affected teeth. Caps were then made to accurately fit the root ends without the ordinary pins passing into the root canals—this being analagous to the caps used for Richmond crowns. Another similar set of caps were made to fit the amputated crowns and having pins passing into the tooth structure. These caps were then placed in approximation with their relative root caps and the two soldered together. The double caps were then placed on the roots, an impression taken, investment made and the entire appliance soldered together as a bridge. This then was cemented in position and the amputated crowns of the natural teeth cemented into the open caps made to receive them. This method requires considerable skill, but when one accomplished, is most satisfactory.

Nearly all men skilled in the treatment of this disease have ideas of their own regarding permanent appliances but the above will serve to give an idea of what is possible.

Now taking up the medicinal treatment together with the mechanical aid that is ever necessary we find that the first step is a thorough and entire removal of all calculi. This is accomplished by the use of suitable scalers of long slender character, so as to avoid undue laceration of marginal tissues. An instrument of the push cut is preferable, although some operators use a draw cut. This, however, is optional, the main object being the thorough removal of deposit and minimum laceration of tissue. Each affected tooth should be completely scaled before another is attacked. The operation may be supplemented by frequent flushing with 3 percent hydrogen dioxide or pyrozone to aid in disinfection, eradication of loosened deposits and pus. Applications of trichloracetic acid, 10 percent, are often valuable in case of tumidity of gums. This has an astringent action and frequently aids in the loosening of deposits. Subsequent to scaling a thorough flushing with pyrozone, 3 percent, followed by applications of a tonic astringent such as iodid of zinc, 20 percent, or 50 percent iodin and alcohol are effective. Hydronapthol is also used in this connection. A good antiseptic and astringent mouthwash should be prescribed for use by the patient. The writer has had good success in a number of cases by use of Lactic acid, full strength, applied to the pockets on pledgets of cotton. Glyco-thymoline has proved a very effective agent in connection with the trouble.

Having pretty thoroughly covered the causes, clinical history, diagnosis and treatment of the first variety of pyorrhea, I wish to call attention to a second variety, commonly designated phagedenic pericementitis. It is this phase of the disease which has given rise to the extensive research by many men and their probable conviction that the exciting cause is a specific bacterium. Dr. Younger, in an able paper read before the New York Academy of Stomatology, in 1899, made the statement that he had made cultures from both, the exudates and calculi of this form of pyorrhea and had actually isolated, grown and developed on agar-agar, gelatin and blood-serum a specific bacterium, to whose action he attributes the disease. As I have before stated, Dr. Miller, of Berlin, has repeatedly found the diplococcus pneumonioe in pyorrhea exudations. However we cannot wholly ignore the calculus theory as bearing distinctly on the cause of this trouble by causing a debilitated or degenerated state in the pericementum.

The predisposing causes may be considered as any influence which tends to produce a degenerated condition of pericementum such as diabetes, anaemia, arthritic diseases or any condition which produces suboxidation. The exciting causes, according to Burchard, may be classed as the overuse, misuse or disuse of the teeth together with calculus deposition.

This disease is generally ushered in by an unexplained shifting of some tooth, or rotating of same. The cause for such an action is not accounted for by any irregularity of occlusion nor marginal inflammation and the patient is unable to explain it. Examination will reveal, in most cases, an absence of gingivitis unless subgingival calculus be present. A general marginal atrophy may, however, be apparent. Calculus on a lateral aspect of the root is found giving rise to pericemental degeneration and pus-formation. This degeneration continues to enlarge, encroaching on the surrounding tissues until we have a localized collection of pus similating an alveolar abscess and taking on the name, primary pericemental abscess. This condition is accompanied by alveolar necrosis and all the attendant symptoms of alveolar abscess. As long as this condition remains localized, it is known as primary abscess, but should this contained pus find its way out, either by way of the root to gum margin or through the alveolus and overlying tissues to gum face, it then developes into what is known as secondary pericemental abscess. In diagnosis the pericemental abscess is often confounded with acute septic apical pericementitis, but the condition can be conclusively diagnosed by ascertaining the vitality of the pulp.

It is thoroughly evident that this variety, like the first, is a disease of the pericementum, accompanied by alveolar necrosis and yet the two are quite different from a diagnostic standpoint. Marginal gingivitis is almost always absent in the second variety and often sub-gingival calculus. Fistulous openings, malocclusions and malposition of affected teeth are general acompaniments. The pockets are deep, extending directly toward the apical aspect of the root and the deposits are difficult of recognition.

The treatment of the second variety is essentially the same as that of the first variety, although the result is, as a rule, not so successful or promising. The all-important factor is, of course, thorough and complete removal of deposits and the obtaining on an aseptic condition in and about the affected parts. The use of antiseptic agents would be followed by the tonics for regeneration of tissue. Dr. Younger is a great advocate of the use of lactic acid, full strength, in pyorrhea pockets. He claims that it is not a solvent for the calculus as many have claimed, but that it is a stimulating tonic very active in the production of granulations. There is a proprietary preparation on the market known as acetezone which is a benzoyl acetyl peroxid and possesses great antiseptic and disinfectant properties. Dr. Wheeler of New York City recommends it very highly for treatment of this condition.

It was the writer's privilege to assist in the treatment of a case of phadenic pericementitis in August, 1903, which case presented affection of the six anterior superior teeth. Fistulous openings were present over the four incisors large enough to insert the end of an ordinary lead pencil. It was necessary to remove a larger part of the labial plate of the alveolus, due to its necrotic condition, thus exposing a portion of the roots to view through the openings in the gums. Trichloracetic acid was used on the margins of the fistulae to cauterize all necrotic tissue, the roots were thoroughly scaled and the pockets and fistulous openings flushed first with pyrozone, 3 percent, and later with glyco-thymoline full strength. The teeth were supported by splint and pockets treated with lactic acid, full strength, and the fistulous openings packed with antiseptic gauze. Granulation rapidly formed and within two weeks the teeth had tightened visibly, and eventually the case was dismissed with the teeth in situ and in apparent health.

Prophylaxis again enters into the treatment of this variety as with the first. Too much stress cannot be laid upon the necessity of frequent examinations for removal of any collected calculus and the application of antiseptic treatment. Where splints are required, those described in connection with the first variety are available.

There is a third variety of pericemental inflammation which is by some classed under pyorrhea alveolaris and known as gouty pericementitis. This condition is due largely to systemic disturbances, however, which brings about a deposition of uratic salts in the pericementum by the blood. The pathology is essentially the same as that of gout—there being present a deposit which exhibits a combined reaction of urates and calcium phosphate brought by the blood to the joint or articulation between tooth and socket, there setting up an inflammation followed by degeneration of pericementum and alveolus. This condition is of rare occurance in patients under thirty-five years of age. The disease is not as rapid in development as the other varieties of pyorrhea already discussed, nor does it affect many teeth at one time. Usually one or two teeth only are affected. Subsequently symptoms may appear in other teeth singly but they may escape attack for years.

This condition is apparent from a diagnostic standpoint only when seen in its later stages. Some one tooth becomes sore and tender upon percussion, the gums overlying the apical portion of the root exhibits evidences of underlying inflammation—a swelling may occur at this point which, when opened into exudes, a glairy, mucous-like discharge, differing materially from pus. Examination of the underlying structure reveals a disappearance of a portion of the alveolar wall, a denudation of the pericementum on the exposed root and the presence of rough calcarious bodies. Sometimes the symptoms may prevail without the swelling at the apical portion of the root, but there will be evidence of the glairy mucous-like discharge at the gum margin. Of course the affected tooth is visibly loosened and tenderness to percussion prevails.

Local therapeutics in this condition will relieve in part the disagreeable symptoms but will not cure. If the condition is seen early the anti-gout treatment affords marked relief. The local treatment is essentially the same as for phagedenic pericementitis before described but is of little avail unless attention is paid to general or systemic treatment at the same time.

The general treatment includes of course regulation of diet and medicinal agents which will eliminate the gout poison and prevent its formation—diuresis, of course, being the general underlying principal of such therapeutics.

I might say that this condition of gouty pericementitis almost invariably has a history of hereditary or acquired gouty conditions, and yet the trouble may make its appearance unprecursed by any such history.

This condition is not directly parallel in respect to causes with the other varieties heretofore described, but is generally classed under the head of pyorrhea and hence is briefly described herein.

In conclusion I would say that although the prime causative element of pyorrhea proper has not yet been universally established as due to a specific bacterium it is strongly advocated by many leading pathologists and undoubtedly the validity of it will be conclusively proven in the near future, thus bringing us to a more definite line of action in regard to its prophylaxis and subsequently a lessening of its univerasl frequency.

DISCUSSION OF DR. SPALDING AND DR. BURBRIDGE PAPERS.

DR. CAMPBELL: I would like to ask Dr. Burbridge whether he ever succeeds in restoring the lost tissue in bad cases of pyorrhea.

DR. BURBRIDGE: I would say that it is as impossible to restore lost gum tissue as it is to restore an arm which has been cut off. It is impossible to grow a new arm if a man loses it, and so also it is impossible to grow new bone or gum tissue. It, however, is possible to restore the gums to somewhat of their original condition. However, the gum will never be restored to its original position on the teeth after having been sloughed away by the process of inflammation. The same thing holds true here as in the case of an arm amputation.

DR. WALTER MCKINNEY: I had a case a short time ago which seemed to be only a case of pyorrhea, but after the teeth were extracted it assumed the form of alveolar necrosis. I would like to ask if people who work in match factories have pyorrhea without necrosis of the jaw?

Dr. Burbridge: Phosphorus poisoning is a distinct condition. It may simulate in a way the condition of pyorrhea, but it is a distinct disease. It may be possible that the bone necrosis which is due to phosphorus poisoning may cause necrosis of the gum overlying it.

Dr. J. Q. Byram: I think the time has come when we, as a profession, should get the idea out of our system that we are "cleaning teeth." It is so common for a patient to come to us and hear them say "I want my teeth cleaned." As long as we do that work only, we can hardly expect our patients to appreciate this new form of treatment. The time has arrived when it is necessary for us to try to prevent disease. If we can do this by prophylaxis, as we are pleased to call it, it is up to us, as a profession, to begin to practice prophylaxis. I heard a few remarks to-day on the steamer to the effect that we had too much cleaning of teeth in the clinic here yesterday, and it leads me to believe that many of us are not appreciating this new line of work as we should. The trouble with us is that we are looking only for the work that comes to us in the way of filling, or in crowns and bridges; and when a patient presents himself we fill the cavities we find, and carelessly remove any small amount of obtrusive deposit from the teeth. When I say a small amount, I say it advisedly, because there are few operators who take the necessary precautions to thoroughly polish a set of teeth. I believe that we, as a profession, are largely responsible for our patient's indifference to this condition. We remove the more obvious deposits and collect "two" or "three" dollars, or whatever the fee may be, and that is as far as we go. We dismiss our patients without endeavoring to educate them to the point where we may be able to have them often enough to thoroughly polish and care for their teeth. I believe that our work in the future will be along this line, and that it will be the principal work of most of us to try and prevent caries, rather than to fill the teeth after they have become diseased. I am frank to say that I was rather surprised when I saw Dr. Spalding's name on the program of this paper. I have never had the good fortune to meet Dr. Spalding personally until yesterday, although I have known of him for a number of years, and I rather thought that he would be like some of the rest of us probably, give all his attention to this branch of work and neglect other things. I am frank to say that I am not in this line of work as I hope to be, and after witnessing the clinics yesterday, I wish to say that they gave me new inspiration and enthusiasm, and from the benefits I received yesterday, I hope to go home and be able to practice this branch of dentistry in a more scientific manner.

Dr. Wood: It has taken me three years to come to a point where I can fully appreciate the line of prophylaxis work as laid down by Dr. Smith, to my own satisfaction, and get where I can bring about satisfactory results. Now as to the paper on pyorrhea alveolaris, Dr. Burbridge has, without question, given this subject a great deal of attention and he has given us an excellent summary. I have tried to alleviate and bring about the cure of these cases for several years and with more or less unsatisfactory results; only within the last year or so have I had cases of positive proof of cures. I have had in my practice during the last year positive proof of cure of teeth badly affected with pyorrhea alveolaris; teeth have become firm in the process and the gum restored in a measure after having been denuded for more than half the length of the root of the tooth. I am well satisfied to undertake treatment in very bad cases. I speak this way because we hear men say every day that they do not believe it is possible to do any good in the treatment of most cases. It is only because they have not been enthusiastic enough to follow it to a finish and do their best as they have been instructed to do. But, after we do all this, I do not believe that they will stay cured unless we follow up this prophylaxis treatment as given us to-night by Dr. Spalding. The regular polishing of the surfaces of the teeth as accomplished by the hand polishing, I believe an absolute necessity. To be sure, this is a very hard matter to get the patient to understand and appreciate enough to come back regularly. One of my most important cases which I believed the treatment to be most necessary, recently dropped me "cold" because I coaxed her to come back every month. She stuck to it a few months and then quit. This is very discouraging, and I do not know what to do with her, but I am not going to quit. I believe that this work should be thoroughly taught in our college clinics. The colleges have been very lax even in scaling the teeth. The average student in the colleges the country over is taken to the chair and the patient is placed in the chair, and the professor says: "Clean this set of teeth." The professor goes to the other end of the room and the student slings his brushes at the teeth and gets the patient out of the room at the earliest opportunity. The student considers cleaning teeth a menial work and does not realize the importance of the operation. The colleges must take this up vigorously, and I believe a good many of them are at it at the present time. We have good evidence that the University is doing it, as we saw Dr. Hoff working so enthusiastically yesterday. I believe to accomplish anything in this prophylaxis treatment we must persevere and enthusiastic. I believe it is the most important object of practice to-day. I believe that the man who does not take it up and follow it closely is going to be left way in the rear very soon. I do not believe it is possible for any dentist to do this until he allows his own mouth to be subjected to that treatment for a time regularly, until his own mouth is put in good condition he does not know what it means.

For a dentist to stand behind a chair and advise his patient to carry out this treatment every month, when he himself has a foul mouth, is rediculous. I would advise any man who has an idea of taking up this work to go to his nearest neighbor dentist, that he can get to do it, and have his teeth treated by these methods thoroughly, and then he will be in a position to do something for his patients.

Dr. W. G. Shattuck: I have had some experience in treating pyorrhea, and I find in the early stages the destroying of the pulp has been very beneficial. If there is any one thing in the student's college life that he wants to get rid of, it is cleaning teeth; they are all after gold fillings, they think it is menial mechanical work. Cleaning teeth is one of the most important things we have to do. I am doing the best I can to get our students to realize this fact, so as to stimulate them to do it. I tell them that they may in this way find cavities that need to be filled with gold. If this meeting can create an interest in this subject in the colleges, I am willing to work hard to have it come about. Students do not realize that we have got to treat pyorrhea, they say, "Oh, it is not curable." I would stand for one to urge on this treatment.

Dr. E. A. Honey: I want to congratulate Dr. Wood on his mention of the fact that this is work we should begin in the colleges, and I want to say that it is my experience in examining the mouths of my patients that they do not all need gold fillings, but nearly every mouth is in need of prophylaxis. Now, if your students are to be marked on prophylaxis just the same as they are marked on the quality of their gold fillings, they will see to it that their mark on prophylaxis is up to the point that they are marked on gold fillings. We see mouths from all ages, from the child of six years to the man of eighty, and with very few exceptions they are in need of prophylaxis, and many of them may not be in need of gold fillings. I would insist, with Dr. Wood, that it is up to the colleges to educate our future dentists to this standard of prophylaxis, that many in the profession are individually trying to educate themselves.

Dr. N. S. Hoff: This, it seems to me, is one of the most important subjects that could come up for discussion, and I am pleased to believe that the subject will never be presented to the profession in any better form than it has been presented here. The statement that Dr. Honey just made leads me to say that the colleges are not the only people that need to get the prophylaxis religion. I

went to the Central Michigan Dental Society recently and gave a clinic on prophylaxis. The members of that society passed around to see my clinic as they did the others; they would look and say, "What is he doing?" Some one would answer, "He is cleaning teeth," then they would pass along. I had a dentist in the chair at the time, I knew I was sure of him, and I persuaded two other dentists to stay at that chair with me for two hours. Those three men, I am pleased to say, are converts to prophylaxis and are working at it. Shortly afterwards I went to Kalamazoo to attend the Southwestern meeting, where I had a similar experience, excepting that I got five men with me, one of whom I had in the chair there also. By this means, I think, those men were inoculated with prophylaxis. I came here yesterday and had five or six young girls come to me and want to have their teeth cleaned. I said, No, I have no time. I hunted up a dentist and finally got one in my chair and gave him a prophylaxis treatment, and now he is a convert, and he told me to-night that I had cured him of a very severe case of dysentery; he says he is a well man to-day whereas he was sick when he came to the city, and he attributes it to the treatment I gave him yesterday.

I present these facts and you can draw your own conclusions. I myself got into this prophylaxis business in the same way. I got a dentist to treat my mouth. I never got particularly interested in it until I got some one at my own mouth, and since I have had that work done in my own mouth there is not a patient that gets into my chair now but what gets prophylaxis, and some of my patients that I have treated before when they come to me now, have said to me: "What are you doing?" I answer, "I am giving your teeth a treatment which they very seriously need." They usually say: "I have always thought you were very thorough in cleaning my teeth, but you have never cleaned them that way before." I say: "No, I never did, but they should be cleaned that way once a month hereafter." I feel that we should devote more of our time to this work, as I believe that we can do our patients more good in this way than in any other way. I believe this treatment is needed more in dentistry to-day than anything else that is done; there is no conservative effort that is so important and there is no one thing that would do the public so much good as this prophylaxis treatment. Some one said here to-night, that there is hardly a mouth but what needs this work. I would say, there is almost no mouth that comes into your chair but what needs this work and the majority need it seriously. I think it is high time that our practitioners were taking this matter more seriously. If you will consider the account of my experience with dentists and also the statement that others have made here on the floor to-night, you will believe with me that the colleges and students are not alone at fault. You may not like to clean teeth, but prophylaxis is a different thing. Dr. Burbridge has given us an excellent paper on the subject of pyorrhea. I do not think I have ever heard a better one. Your text books will not give you a better description of the etiology of this subject. He has very classically outlined the etiology of this disease. If you will take that paper and study it, it seems to me that you will be in possession of the knowledge for outlining for yourself the different treatments of this disease. He has called your attention to the different conditions from the mere irritation of extraneous deposits to hopeless necrosis. Each of the conditions need different treatment. We have been in the habit of trying to apply the same specific treatment to all these conditions, and we have been forced to conclude that some cases of pyorrhea are incurable. I do not believe there are as many hopeless cases of pyorrhea as we are inclined to think. Many of the cases of pyorrhea that we now pronounce incurable, I believe are curable on a prophylaxis basis. They are almost all infectious, but very slight applications of the usual disinfecting remedies, early employed, will put these cases in a condition which will be very materially helped on by the prophylaxis treatment. In extreme cases of necrosis, of course, we have to operate.

We should become intelligent as to conditions and apply such treatment as will put the structures into the form which we can treat best by prophylactic means. It looks to me as if we are just on the eve of a revolution in the treatment of oral diseases, and I predict that within the next two or three years that we shall treat pyorrhea successfully. Too many think they are too busy making plates, and crowns, and inlays to take up this work. Such work is good in its place, but we must not give prophylaxis any second place. It is needed in almost every mouth and is fundamental to the health of every patient, and should be given a first place.

Dr. Smith: If the name "prophylaxis" is going to make more men clean teeth, then I am glad we have a new name for it. For a good many years it has been the most important work I have done. It was simply a method of going over the teeth and thoroughly keeping them clean—a work that I know is generally neglected by dentists. While many dentists are capable of doing fancy operations, they are neglecting their cases by neglecting to have their patients keep their teeth perfectly clean. I am going to give the dentists of my town a talk on prophylaxis when I get home. To my mind, prophylaxis is a thorough cleaning of the teeth, but I like the new name.

A Member: I took the State Board Examination recently, at Ann Arbor, and I was given such pointers on cleaning the teeth that I have heen cleaning teeth better and appreciate that part of my practice more than ever before. I think if practiced more scientifically it will be appreciated, and will become more of a pleasure and less of drudgery. It becomes more interesting the more attention we give to the details, and to getting our office and instrument in such condition that we can handle these cases conveniently.

Dr. G. C. Bowles: Some of the speakers have said the colleges are not to blame because their students will not clean teeth. Perhaps they have not been in the past, but if the students do not clean teeth in the future, they will be to blame. Dr. Smith says from 70 to 90 percent of caries can be prevented by prophylactic treatment. He has also demonstrated that by this prophylactic method pyorrhea can be prevented absolutely. Then there is nothing so important to the profession generally as to get this one thing thoroughly impressed, and should we get this thoroughly impressed upon our minds at this time, this meeting will have been successful. We had a little illustration of the attitude of the profession toward this thing during the past year, in February; our city society voted to invite Dr. Smith to come here to demonstrate. At that time I voted against the proposition, as I thought I knew how to clean teeth. I thought any one could do that. We did not happen to get Dr. Smith. Next year we will be able to make a success of the clinic for two days in having Dr. Smith. In the first paper I noticed that the essayist laid emphasis on prophylaxis. That was Dr. Smith's term, and he had in mind Dr. Smith's method of course. In the pamphlet Dr. Smith has on prophylaxis I noticed the etiological points that the essayist considers. Some say this is not pyorrhea. Pyorrhea, he says, is entirely local, and is not systemic: and a good many other things that have been characterized by a great many terms, but is simply due to local poisoning, and that the care of the teeth will prevent disease. Burchard and others have discussed this subject in their books, and state that they are not always successful; but Dr. Smith is positive in his dogmatism. If Dr. Smith is on the right ground, cleaning teeth, which we have always treated so lightly, is the supreme thing. I have been practicing prophylaxis since last February, and will say that I am converted, and I think if others who are finding fault with teeth cleaning will get to work for the next six months, they will never find fault again. Dr Spalding says this treatment will check erosion. I wonder if it really will. Another point which has occurred to me is that in mouths that are the worst affected by pyorrhea we usually find the least decay, so many of the teeth that we have to sacrifice are free from caries. I can agree with him that in mouths possessing these affections, mouths where the micro-organisms were so abundant that pyorrhea had gained to such an extent that the teeth had to be sacrificed, although caries was not present.

Dr. M. T. Watson: There is no field of dentistry in which I am more interested at the present time than the development of the field of prophylaxis, and I am going to relate to you briefly a personal experience. Some months ago Dr. Spalding called me up and asked me if I would put myself under his care for a few weeks that I might discuss a paper that he was to read for the local dental society some weeks ago. I was very glad to do so, as I thought I would have a joke on Dr. Spalding, for I have prided myself on being cleanly in my habits. The first time I went over to Dr. Spalding's office I expected to spend a little time chatting with him and have him send me back and say I was not the kind of man he was looking for. He did, however, remark casually that my mouth was in a remarkably healthy state, but, greatly to my surprise, he went to work on it. For the next six months I went once a week and had him care for my teeth. I believed I was taking good care of my teeth and that they were free from accumulation of soft masses, and that my mouth was reasonably free from decay, but my gums were sensitive and certain buccal surfaces of my teeth were sensitive. I do not believe that they were due to any inattention to the teeth. After a few weeks of treatment, instead of using a soft brush I could use, with perfect comfort, a brush with an unbleached bristle. The comfort that I have felt since my mouth was put in that condition, and I am able to keep it free from the soft deposits, and the change in condition of my gums has converted me absolutely. I want to say that in my judgment it is equally as important to restore mouths to a healthy condition as it is to keep mouths in a healthy condition.

I was unfortunately detained to-night so that I only heard the latter part of Dr. Burbridge's paper, and one question arises in my mind: In the third variety of pyorrhea alveolaris he stated that it was associated with a systemic condition, and the thought occurred to me: If the primary cause of this variety is a systemic condition, why does it affect only one, two or three teeth. One cannot help wondering why more teeth are not affected if the primary cause is systemic. I cannot understand why some particular teeth were otherwise in a healthy condition if they could be more readily affected, but we see mouths where the teeth are in fine condition as far as caries, etc., is concerned; what, then, can be the reason for this particular variety affecting only one or two teeth?

Dr. E. T. Loeffler: I want to say that this work has been carried on at the college, and is largely under my direction, and I am sure that the members of this association can rest assured that by the end of next June strict attention will have been given to encouraging students to do this work. It is a fact that it is difficult to interest students in this kind of work, but we shall strive to do the best we can.

A Member: I would like to ask whether pyorrhea is associated with persons subjected to uric acid conditions. I have noticed in my practice most of the cases are of those who are subjected to stomach trouble and uric acid conditions—a condition that it seems to me comes from indigestion, rheumatism, uric acid headaches. Most all of these cases who suffer from pyorrhea have more or less indigestion and rheumatism.

Dr. C. P. Wood: For some years I had a general understanding that all these bad cases of pyorrhea must be associated with uric acid systemic conditions, rheumatism, etc. I have had it all knocked out of me within the last year or so. I have inquired especially of those cases showing any symptoms of that condition, rheumatics, acid conditions or other stomach troubles—no stomach trouble at all is the answer time and time again. I say again, the great majority of these cases have said they never had a touch of rheumatics, and I think it is just as it happens if those cases are associated with rheumatic trouble.

Dr. C. H. Worboys: I think we had all better take a lesson in prophylaxis. The very first thing for us to do is to find out what instruments to buy and use. In half of our cases, I presume we have not a fit set of instruments to clean a set of teeth with. We have instruments to clean every part of the teeth that the patient can see, and shine them up so they will look well for the next party, but when it comes to removing those little particles of tartar away below the gum, how many of us have the proper instruments. If we have them, how many times have we used them successfully and removed these particles that you can hardly see when you get to them. Besides that, how many of us really know how to use the other instruments, the hand instruments, polishing the roots down under the gum; but you all know that if you don't do that you will not get any results and will not be successful. With the pyorrhea, I throw up both hands. I do not know how to do it yet. It is not the fault of the tissue, it is not the fault of the disease—it is my fault, I do not know how. I say I do not know how, because I don't get results, and there are some fellows who do get results, and it is because they know how. I think that is the way with the most of us. I am doing something. It is not exactly along the line of prophylaxis, but it is in the way of advice to my patients and will help along that line, and that is to encourage the very best development of the permanent teeth, and that by the strenuous use of the deciduous teeth, and if we can teach parents to have the child keep the deciduous teeth clean, we are making a good long step toward the welfare of the adult. Unless we go back to the thorough use of these deciduous teeth so that they can grow up strong with their sulci united, or the bones of development united at the Suters, and be peramnent, we will not always have use for prophylaxis, because the teeth will be so decayed before we can get hold of them that there will be nothing to do. I believe that the thorough use of the deciduous teeth is the only way to stimulate the perfect development of the permanent, and if we can have perfectly developed teeth, and then keep them in a prophylactic condition so that they can be thoroughly used, I do not think we will have very much pyorrhea, and yet we find we have pyorrhea in those mouths where the perfect teeth are, whether this is from lack of thorough use of them I do not know. I believe that the muscles of the jaw are not powerful enough to crack, on any food that the human being wants to eat, a perfectly developed tooth that is not decayed.

Dr. C. A. Burbridge: There were a few questions asked. One was why it was that only one tooth should be affected. I do not know that I can explain it satisfactorily, because it is hard to explain. I said that the pathology of the third variety was the same as the pathology of gout. We know that gout, as a rule, affects only one joint. Just why the disposition of uric salts should be directed to that point is hard to tell, and why the determination of blood loaded with uric salts should go to the pericemental tissues of one tooth is also hard to explain. It is found, however, that such is the case. Another questioned concerning the association of pyorrhea with uric acid diathesis. It was thought for a number of years before this subject was gone into thoroughly that pyorrhea was always associated with patients of the uric acid diathesis. That, however, has to a large extent gone out of vogue. I, for myself, am a great advocate of the theory that pyorrhea is due to a specific bacterium, and right in line with this subject of prophylaxis. It appears to us that perhaps it may be due to a specific bacterium, because the position in which food materials and degenerated tissues are always found. This slime found about the necks of the teeth furnishes an excellent media for the proliferation of bacteria, and it is legitimate to think that the disease should be caused by a specific bacterium. It often happens, however, that cases of pyorrhea are associated with persons troubled with stomach trouble, rheumatism, etc., but we hardly think it is due to that.

Dr. E. B. Spalding: I think the reason that the dentists do not care to clean teeth, as they call it, is because they do not know what they are doing. It requires success to make us enthusiastic in anything that we undertake. Now if we do not know what results we are getting or what results we will produce, or have not had experience with it, we will not attempt a thing as a rule. If the dentist appreciates what he is doing in giving prophylaxis treatment it will be different. In prophylaxis treatment there are coatings on the teeth which neither the dentist nor patient can see, and the only way to know that these are there is by the sense of touch, and that sense of touch has to be cultivated. At first we want to know why the engine is not used. The engine will not accomplish it in the first place because you would not know what you are doing with the engine, as you can have no sense of touch through the heavy hand piece. Some say: "Why don't you use the engine for removing most of the deposit first?" When you first begin to put in a filling in college, you are told if you take care of the margins the center of the cavity will take care of itself. If we remove from the surface of the tooth out of the way, the broad exposure of tooth surface will be taken care of naturally. The use of the engine irritates the gums. Now that we have taken it for granted that there is something in prophylaxis, we should go at it with hand methods and take one case and treat it often enough by hand work, and treat it every week. It is better to treat a fellow dentist. The first thing you know you have discovered a lot of things that you did not know before. I am afraid that a great many men will take up what they call cleaning teeth and call it prophylaxis, and they will not know what they have produced and they will do it once, and probably after six months or so they will do it again. Prophylaxis cannot be done unless it is done at least once a month. Of course some will say: "I cannot make my patients come." A patient will come and get treatment, and pay for it, if he knows what he is getting. If the dentist does not know what he is doing, and does not explain it to the patient, the patient will not pay him for his time.

One point that I am surprised has not been brought up, and that is that the friction wears the enamel. For my own satisfaction I figured out in treating a case that has been under supervision for some time, how much time would be devoted to a single tooth in the mouth. I find that a minute of time is abundant for any one particular tooth. Now, if that is done once a month, that would mean 12 minutes a year, that will be an hour's time in five years; in twenty-five years, you will devote twenty-five hour's time. I have put upon the labial surface of one tooth only, about one and one-half hour's time, polishing with a stick and pumice stone, as I would in the mouth on that one tooth, and I defy anybody to find that there is any noticeable wear on that tooth. In the mouth there are many extraneous coatings. These coatings are the bacterial films. As it takes two-thirds the amount of time that you are using on the tooth to remove those coatings first, you are only giving about one third of the time to the polishing of that enamel. Out of the mouth you are polishing that tooth twenty seconds out of the minute with the grinding material.

As to the colleges teaching students. The students do not want to do the work because they do not know its importance, and they do not know because they have not been taught, and they have not been taught because the instructors do not know. If the instructors will equip themselves, they will get the students to devote their time to it likewise. If once a week each student devoted some time to it, at the end of a year the student will be a graduate to prophylaxis. If we use a sufficient amount of oral prophylaxis we will not have to put in many gold fillings.

In regard to children, we should begin early, as soon as they have teeth and you can do anything for them in the chair, because it is as important to care for their teeth and keep them from suffering pain, and also teach them that the dentist is not a brute as the majority of them think, then they are glad to come to see him and enjoy it, and when he does hurt them, they will bear the pain and think he is not such a terrible fellow after all.

Prophylaxis is something that we cannot learn in five minutes all that should be known about it, because it takes time to develop a proper sense of touch with the stick, or instrument, but if we will take a single case and keep at it for a time we will, I am sure, learn enough to take it up among our own cases. Dr. Bowles asked about erosion. Erosion is about the most disheartening thing about the whole subject. I saw a bad case of erosion with Dr. Rhine which he said had been entirely arrested for some years, and it was through this work entirely. I have had a number of cases that I have been treating, but the trouble is not inflammation that you are treating; you can see the change, but where there is erosion you cannot see the new substance grow. Such irritation is the hardest to subdue that I have taken hold of, but I have succeeded in making a marked change in it. Some one spoke of stomach trouble in connection with pyorrhea. I would like to relate a case that I saw in Dr. Smith's office this last year. This man told me this himself. He is a resident of Philadelphia, a wealthy business man. He said a year ago he was on the point of jumping into the Delaware river. He said: "I was so anaemic, had stomach trouble, was out of health and nervous in every way, had been to every stomach specialist I could hear of, and a number of dentists for pyorrhea, but no one helped me. Some one suggested that I see Dr. Smith." Dr. Smith took some models of that case so that it could be compared. This man said: "I came to him and he gave me a treatment." I might say that Dr. Smith is not as careful sometimes in the matter of causing his patients pain as some men are. He probably went for this patient and gave him a pretty severe treatment. The patient said: "I went out of that office, it had hurt me so that I swore I would not go back again." However, he kept at it, and he said to me: "Today I am a changed man in the whole matter of my life." He asked Dr. Smith what he should not eat. He said: "I have been denied beef, strawberries, beer—almost everything except white meat, fowl and plain foods. Dr. Smith said: "Eat anything you want," and he did. The patient said to me "I soon found that he was taking out of my mouth something that was going into my stomach and making this trouble."

You will find that in cases of pyorrhea the teeth are coated and filthy and there is a foul odor, and if you will keep that off you will find that the proper scaling of the pockets, which is most essential and the inflammation in the gingiva will subside very rapidly, and it is on record that a remarkable number of cases of stomach trouble are due entirely to the mouth infection. We little realize what is going into our stomachs every time we take a mouthful of food and wipe off some of this infection.

